# Murine *Fig4* is dispensable for muscle development but required for muscle function

**DOI:** 10.1186/2044-5040-3-21

**Published:** 2013-09-01

**Authors:** Aaron Reifler, Guy M Lenk, Xingli Li, Linda Groom, Susan V Brooks, Desmond Wilson, Michyla Bowerson, Robert T Dirksen, Miriam H Meisler, James J Dowling

**Affiliations:** 1 Department of Pediatrics, University of Michigan Medical Center, Ann Arbor, MI 48109-2200, USA; 2 Department of Human Genetics, University of Michigan Medical Center, Ann Arbor, MI 48109-2200, USA; 3 Department of Pharmacology and Physiology, University of Rochester Medical Center, Rochester, NY 14642, USA; 4 Molecular and Integrative Physiology, University of Michigan Medical Center, Ann Arbor, MI 48109-2200, USA; 5 Neuroscience Graduate Program, University of Michigan, Ann Arbor, MI 48109-2200, USA

**Keywords:** Autophagy, Congenital myopathies, FIG4, MTM1, Phosphatidylinositol

## Abstract

**Background:**

Phosphatidylinositol phosphates (PIPs) are low-abundance phospholipids that participate in a range of cellular processes, including cell migration and membrane traffic. PIP levels and subcellular distribution are regulated by a series of lipid kinases and phosphatases. In skeletal muscle, PIPs and their enzymatic regulators serve critically important functions exemplified by mutations of the PIP phosphatase *MTM1* in myotubular myopathy (MTM), a severe muscle disease characterized by impaired muscle structure and abnormal excitation–contraction coupling. FIG4 functions as a PIP phosphatase that participates in both the synthesis and breakdown of phosphatidylinositol 3,5-bisphosphate (PI(3,5)P_2_). Mutation of *FIG4* results in a severe neurodegenerative disorder in mice and a progressive peripheral polyneuropathy in humans. The effect of *FIG4* mutation on skeletal muscle has yet to be examined.

**Methods:**

Herein we characterize the impact of FIG4 on skeletal muscle development and function using the spontaneously occurring mouse mutant pale tremor (*plt*), a mouse line with a loss of function mutation in *Fig4*.

**Results:**

In *plt* mice, we characterized abnormalities in skeletal muscle, including reduced muscle size and specific force generation. We also uncovered ultrastructural abnormalities and increased programmed cell death. Conversely, we detected no structural or functional abnormalities to suggest impairment of excitation–contraction coupling, a process previously shown to be influenced by PI(3,5)P_2_ levels. Conditional rescue of *Fig4* mutation in neurons prevented overt muscle weakness and the development of obvious muscle abnormalities, suggesting that the changes observed in the *plt* mice were primarily related to denervation of skeletal muscle. On the basis of the ability of reduced FIG4 levels to rescue aspects of Mtmr2-dependent neuropathy, we evaluated the effect of *Fig4* haploinsufficiency on the myopathy of *Mtm1*-knockout mice. Male mice with a compound *Fig4*^+/−^/*Mtm1*^–/Y^ genotype displayed no improvements in muscle histology, muscle size or overall survival, indicating that FIG4 reduction does not ameliorate the *Mtm1*-knockout phenotype.

**Conclusions:**

Overall, these data indicate that loss of *Fig4* impairs skeletal muscle function but does not significantly affect its structural development.

## Background

Phosphatidylinositol phosphates (PIPs) are low-abundance phospholipids that are implicated as regulators of a range of cellular processes, including cell migration, subcellular organelle trafficking and autophagy [1,2]. Phosphatidylinositol 3,5-bisphosphate, or PI(3,5)P_2_, is a low-abundance PIP whose function has recently come into focus [3]. PI(3,5)P_2_ is generated by the action of PIKfyve, a five-position phosphoinositide kinase [4]. PI(3,5)P_2_ is metabolized by FIG4 (Sac3), a five-position phosphoinositide phosphatase, to regenerate phosphatidylinositol 3-phosphate (PI(3)P), and by myotubularins, a family of three-position phosphatases to generate phosphatidylinositol 5-phosphate (or PI(5)P) [5-8]. Of note, maximal function of PIKfyve requires a complex of proteins that includes VAC14 and FIG4 [9-11]. Reduced abundance of FIG4 protein destabilizes PIKfyve, resulting in a threefold reduction of PI(3,5)P_2_ relative to total PI levels [12].

We recently identified and characterized a spontaneous mouse mutant (“pale tremor” or *plt*) with a homozygous recessive loss-of-function mutation in the *Fig4* gene [12]. *plt* mice exhibit severe and progressive neurodegeneration that involves both neurons and glia of the central and peripheral nervous system [13]. The main subcellular phenotype is increased vacuolization due to defective lysosomal function and impaired autophagy [14,15]. In addition, we (the Meisler group) have identified recessive *FIG4* mutations in patients with hereditary peripheral neuropathy (Charcot-Marie-Tooth Disease type 4J) [16-18] and motor neuron disease (amyotrophic lateral sclerosis, or ALS) [19]. However, the role of FIG4 in skeletal muscle has yet to be extensively examined in detail.

Studies of myotubularins indicate a potentially important role for PI(3,5)P_2_ regulation in skeletal muscle [20-22]. The mammalian myotubularin gene family contains 15 members encoding phosphatases that primarily dephosphorylate PI(3)P and PI(3,5)P_2_[23]. Mutation of *MTM1*, the canonical member of this gene family, results in increased levels of PI(3)P, and possibly PI(3,5)P_2_, in skeletal muscle and causes myotubular myopathy (MTM), a severe congenital muscle disease associated with altered muscle structure and profound muscle weakness [24]. Mutation of *MTM1* compromises multiple aspects of muscle function [25], most notably excitation–contraction coupling (EC coupling) [26], the process by which neuronal stimulation to muscle is translated into calcium-dependent muscle contraction. Specifically, *MTM1* mutations lead to severe abnormalities in the structure of the EC coupling machinery [27].

MTMR14 is another myotubularin family member that is important for muscle function [26]. Loss of MTMR14 function in zebrafish and mice causes aberrant autophagy and impaired EC coupling [26,28]. Unlike MTM1, loss of MTMR14 alters EC coupling without significantly changing the underlying structure of the EC coupling machinery. Direct application of PI(3,5)P_2_ increases calcium release from microsomes containing the intracellular ryanodine receptor 1 calcium release channel (RyR1), a critical component of the EC coupling apparatus. Thus, direct regulation of RyR1-dependent stimulated calcium release may represent one important role of PI(3,5)P_2_ and MTMR14 [29].

Given the potential importance of PI(3,5)P_2_ in skeletal muscle, we sought to understand the impact of FIG4 mutation on muscle development and homeostasis. To address this issue, we examined skeletal muscle structure and function in the *plt*-null mouse model of FIG4 dysfunction. We examined *plt* mice, which die before 2 months of age as a result of progressive neurodegeneration, as well as *plt* mice with restored FIG4 expression in neurons [12,15]. The latter mice have no overt phenotype and survive for more than 18 months. Our data reveal that *Fig4* mutation is associated with skeletal muscle changes (atrophy and increased apoptosis) and impaired muscle force generation, but not with abnormalities in the structure or function of the EC coupling machinery. The changes are likely the consequence of impaired neuronal input because phenotypic rescue is largely provided by neuronal expression of FIG4. In addition, we found that haploinsufficiency of *Fig4* does not ameliorate effects of *Mtm1* mutation in muscle, in contrast to a previous report that haploinsufficiency of *Fig4* rescues the neuropathy associated with *Mtmr2* mutation [30,31]. Together, our results support a requirement for FIG4 in skeletal muscle function, but not a cellular autonomous role in either muscle development or EC coupling.

## Methods

### Animal care and husbandry

All animals were cared for per protocol under the guidance of, and with ethical approval from, the University Committee on Use and Care of Animals (UCUCA) and with the assistance of members of the University of Michigan’s Unit for Laboratory Animal Medicine (ULAM), who carefully monitored the health of the rodent colonies. ULAM maintained proper environmental regulation, including temperature and light cycles, unlimited access to water, appropriate food supply and clean enclosures. The Fig4-null mutation *plt* is maintained on two congenic lines, C57BL/6J.*plt*/+ (N14) and C3H.*plt*/+ (N10) [32]. Experiments were carried out on homozygous *plt*/*plt* F1 mice obtained from crosses between the two congenic strains. Pups were weaned according to standard protocols, and tails were clipped for genotyping.

### Western blot analysis

Western blot analyses were performed using the following antibodies: FIG4 (1:1,000 NeuroMab; UC Davis/NIH NeuroMab Facility, Davis, CA, USA) and glyceraldehyde 3-phosphate dehydrogenase (1:1,000 GAPDH; Millipore, Billerica, MA, USA). Mouse multitissue Western blot antibody was obtained from IMGENEX (San Diego, CA, USA). Protein extracts were established from flash-frozen mouse skeletal muscle and brain using T*-*PER tissue protein extraction reagent and a Dounce tissue homogenizer (Pierce Biotechnology, Rockford, IL, USA). Approximately 50 μg of protein were loaded per sample, resolved by polyacrylamide gel electrophoresis on 11% gels, and transferred to polyvinylidene fluoride. Secondary antibodies were used at 1:2,000 (Santa Cruz Biotechnology, Santa Cruz, CA, USA), blots were developed using electrochemiluminescence reagent (GE Biosciences, Pittsburgh, PA, USA), and bands visualized using the Bio-Rad ChemiDoc XRS+ System illuminator (Bio-Rad Laboratories, Hercules, CA, USA).

### Histopathology

Animals were killed by anesthetic injection, which was followed by cervical dislocation. Tissues were then isolated using sterile surgical methods without the use of laminar flow hoods. Muscle tissue from quadriceps and tibialis anterior muscles was dissected and mounted onto small balsa wood pieces that had previously been frozen with drops of Tissue-Tek O.C.T. compound (Sakura Finetek USA, Torrance, CA, USA) and then semithawed with light friction. The mounted muscle tissue was immediately submerged in a −55°C isopentane bath cooled by liquid nitrogen for flash-freezing.

Muscles were cut into 12-μm cross-sections and mounted on Superfrost Plus slides (Thermo Scientific, Waltham, MA, USA) using a Leica cryostat (Leica Biosystems, Buffalo Grove, IL, USA) at −20°C and dried at room temperature before storage at −80°C. Slides were stained with Mayer’s hematoxylin and eosin (H&E) or succinate dehydrogenase (SDH) following standard protocols and mounted with Permount mounting medium (Thermo Scientific). Apoptotic fibers were visualized with the ApopTag Plus Peroxidase *In Situ* Apoptosis Kit (S7101; Chemicon International/EMD Millipore, Billerica, MA, USA). Photomicrographs were captured using an INFINITY*1* digital camera with eponymous software (Lumenera Corp, Ottawa, ON, Canada) visualized through an Olympus BX43 light microscope (Olympus America, Center Valley, PA, USA).

### Ultrastructural analysis

Immediately following dissection, quadriceps and gastrocnemius muscles were carefully cut into approximately 1-mm × 2-mm fragments and incubated in Karnovsky’s fixative overnight at 4°C. Fixed tissue was brought to the Microscopy and Imaging Laboratory (MIL) Core facility at the University of Michigan for processing. Ultrathin sections were analyzed for orientation, and grids were prepared for use on the Philips CM-100 transmission electron microscope (Koninklijke Philips N.V., Amsterdam, The Netherlands).

### Myocyte isolation

Muscle was dissected from the shoulders and legs of dead mice and placed immediately into sterile phosphate-buffered saline. Muscle was then minced finely with a sterile razor, fully dissociated with a mixture of collagenase type I (0.1%) and trypsin (0.1%) in Ham’s F-12 medium, then incubated at 37°C for approximately 1 h with periodic trituration. Cells were pelleted and resuspended in 1:1 Dulbecco’s Modified Eagle Medium:Ham’s F-12 Nutrient Mixture (DMEM/F-12) (Gibco/Life Technologies, Grand Island, NY, USA) with 20% fetal calf serum (HyClone Laboratories, Logan, UT, USA), then filtered through 70- and 40-μm meshes and plated onto collagen-coated dishes (BD Biosciences, San Jose, CA, USA). Media were changed after a 1-h incubation at 37°C, and recombinant human fibroblast growth factor–basic (AA 10-155, Publication PHG0026; Gibco/Life Technologies) was added to a final concentration of 10 ng/ml. Cells were maintained at 37°C in a 5% CO_2_ atmosphere with daily changes of fresh media. Cells were visualized using Hamamatsu ORCA-R2 camera and software (Hamamatsu Photonics, Hamamatsu-shi, Japan) on a Leica inverted microscope (Leica Microsystems). All experimentwas were performed on passages 2 through 4 myocytes. Terminal deoxynucleotidyl transferase-mediated dUTP nick end labeling (TUNEL) staining was performed per the manufacturer’s recommendations on myocytes grown on coverglass coated with fibronectin and fixed with 4% paraformaldehyde prior to staining.

### Muscle force measurement

Extensor digitorum longus (EDL) and soleus muscles were carefully isolated and removed from anesthetized mice. Muscles were immediately placed into a bath of Krebs mammalian Ringer solution with 0.25 mM tubocurarine chloride maintained at 25°C and bubbled with 95% O_2_ and 5% CO_2_ to stabilize pH at 7.4. Using 5-0 silk suture, the distal tendon of the muscle was attached to a servomotor (model 305B; Aurora Scientific, Aurora, ON, Canada), and the proximal tendon was attached to a force transducer (model BG-50; Kulite Semiconductor Products, Leonia, NJ, USA). Muscles were stimulated by square pulses delivered by two platinum electrodes connected to a high-power biphasic current stimulator (model 701B; Aurora Scientific). A personal computer running custom-designed software (LabVIEW 7.1; National Instruments, Austin, TX, USA) controlled electrical pulse properties and servomotor activity and recorded data from the force transducer. Stimulation voltage and optimal muscle length (*L*_o_) were adjusted to give maximum twitch force [33]. While held at *L*_o_, muscles were subjected to trains of pulses 300 ms in duration for EDL muscles and 900 ms for soleus muscles, with increasing stimulation frequency until maximum isometric tetanic force (*P*_*o*_) was achieved [33]. *L*_o_ was measured with digital calipers, and muscle fiber lengths (*L*_f_) were determined by multiplying *L*_o_ by previously established *L*_f_-to-*L*_o_ ratios of 0.44 for EDL muscle and 0.71 for soleus muscle [33]. Total muscle fiber cross-sectional area (CSA) was estimated by dividing the mass of the muscle by the product of *L*_f_ and 1.06 g/cm^3^, the density of mammalian skeletal muscle. *P*_*o*_ was normalized by CSA to give specific *P*_*o*_.

### Simultaneous measurement of macroscopic L-type Ca^2+^ currents and voltage-gated Ca^2+^ transients in myotubes

Primary cultures of skeletal myotubes were generated from myoblasts derived from wild-type (WT) and *plt* mice as previously described [34]. The whole-cell voltage-clamp technique in conjunction with a Ca^2+^-sensitive dye (fluo-4) was used to simultaneously measure voltage-gated L-type Ca^2+^ currents (L-currents) and intracellular Ca^2+^ transients on individual myotubes from 8- to 11-day-old myotube cultures [34,35]. All voltage-clamp experiments were carried out after an approximately 5-min period of dialysis following establishment of the whole-cell configuration. The external recording solution consisted of 145 mM tetraethylammonium chloride, 10 mM CaCl_2_ and 10 mM 2-[4-(2-hydroxyethyl)piperazine-1-yl]ethanesulfonic acid (HEPES) (pH 7.4). The internal patch pipette solution consisted of 145 mM Cs-aspartate, 10 mM CsCl, 0.1 mM Cs_2_-ethylene glycol tetraacetic acid, 1.2 mM MgCl_2_, 5 mM Mg-ATP, 0.2 mM K_5_-fluo-4 and 10 mM HEPES (pH 7.4). A 1-s prepulse to −30 mV delivered immediately before each test pulse was used to inactivate voltage-gated Na^+^ and T-type Ca^2+^ channels without producing significant L-channel inactivation. L-currents and Ca^2+^ release were subsequently elicited by 200-ms test depolarizations from −50 mV to +70 mV in 10-mV increments and a 10-s interval between each test pulse. Capacitative currents were minimized to about 10% using the capacitance cancelation feature of the patch-clamp amplifier. Remaining linear components were leak-subtracted using a P/3 protocol delivered from a holding potential of −80 mV before each test pulse. Peak L-current magnitude was normalized to cell capacitance (pA/pF), which was plotted as a function of membrane potential (*V*_m_) and fitted as *I* = G_max_ (*V*_m_ – *V*_rev_)/(1 + exp[(*V*_G1/2_ – *V*_m_)/*k*_G_]), where *G*_max_ is the maximal L-channel conductance, *V*_m_ is test potential, *V*_rev_ is extrapolated reversal potential, *V*_G1/2_ is the voltage for half-maximal activation of *G*_max_, and *k*_G_ is a slope factor. Relative changes in intracellular Ca^2+^ during each test depolarization were measured following dialysis with K_5_-fluo-4 salt. Fluo-4-dialyzed myotubes were excited at 480 nm and fluorescence emission measured at 535 nm was digitized at 10 kHz. A computer-controlled shutter was used to eliminate dye illumination during intervals between each test pulse. Relative peak changes in intracellular Ca^2+^ were expressed as ∆F/F ([F_peak_ – F_base_]/F_base_) at the end of each test pulse, plotted as a function of *V*_m_, and fitted according to the equation ∆F/F = (∆F/F_max_)/{1 + exp[(*V*_F1/2_ – *V*_m_)/*k*_F_]}, where (∆F/F)_max_ is the calculated maximal change in fluorescence, *V*_F1/2_ is the voltage for half-maximal activation of (∆F/F)_max_, and *k*_F_ is a slope factor. Pooled current-voltage (*I*-*V*) and fluorescence-voltage (∆F/F-V) data were expressed as means ± SEM. Statistical significance was determined using a two-tailed Student’s *t*-test.

### Statistical analyses

GraphPad Prism software (GraphPad Software, La Jolla, CA, USA) was used to calculate the significance of fiber size differences and TUNEL staining results with unpaired Student’s *t*-tests and one-way analysis of variance with Tukey’s *post hoc* multiple comparison test.

## Results

### FIG4 is expressed in skeletal muscle

**Figure 1:**
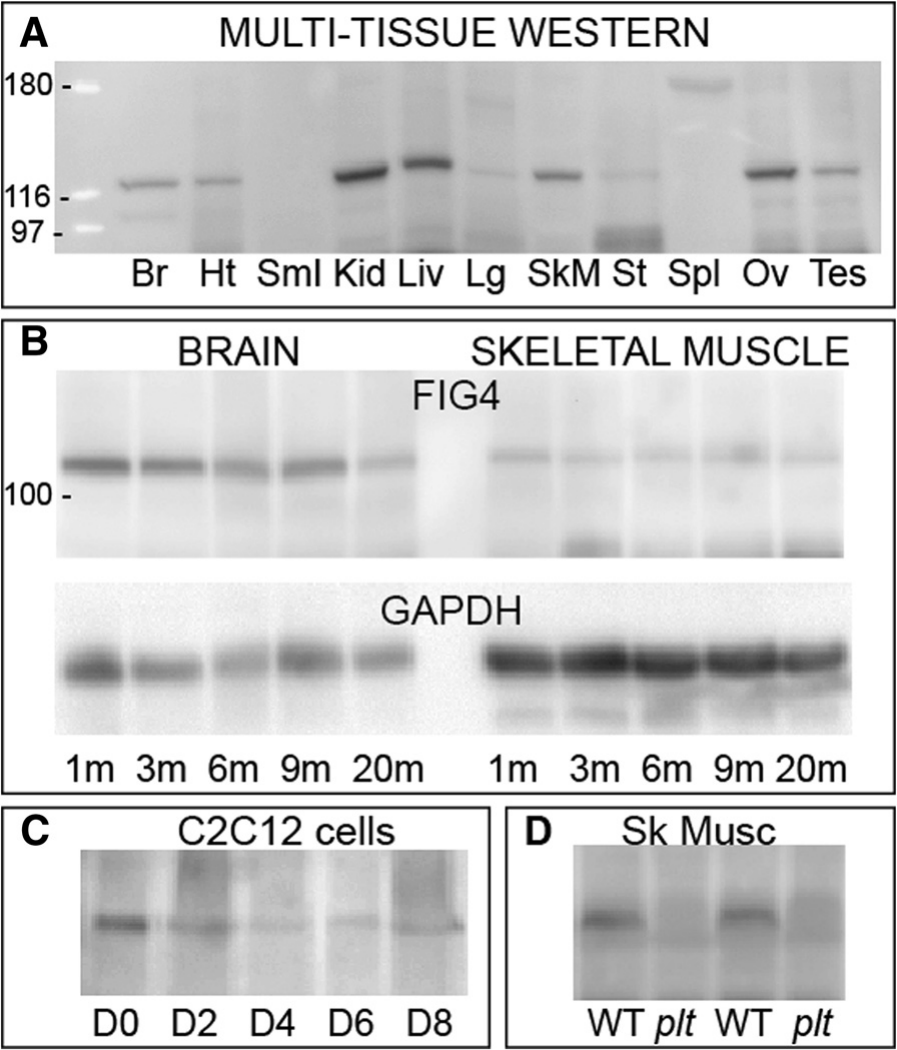
**FIG4 is expressed in skeletal muscle.** Western blot analysis was performed to establish FIG4 expression using anti-FIG4 antibody. **(A)** Mouse multitissue Western blot reveals expression of FIG4 in a variety of tissues (Br = brain, Ht = heart, SmI = small intestine, Kid = kidney, Liv = liver, Lg = lung, SkM = skeletal muscle, St = stomach, Spl = spleen, Ov = ovary, Tes = testis). **(B)** FIG4 is expressed at multiple mouse ages in brain and skeletal muscle. Ages of mice tested were 1, 3, 6, 9 and 20 months. Left lanes are from brain, and right lanes are from quadriceps. The top blot was probed with anti-FIG4, and the bottom blot (loading control) was probed with glyceraldehyde 3-phosphate dehydrogenase (GAPDH). **(C)** Western blot of protein extracts from C2C12 cells at various stages of differentiation. Differentiation was induced by serum withdrawal. Cells were differentiated until long myotubes were obviously present (day 8 = D8). **(D)** Analysis of wild-type littermate and pale tremor (*plt*) mouse skeletal muscle reveals that FIG4 is absent from *plt* muscle.

We wanted to establish that FIG4 protein was present in skeletal muscle. We therefore performed Western blot analysis using anti-FIG4 antibody (NeuroMab). We first used a premade multitissue Western blot antibody (IMGENEX) and found that FIG4 was present in most tissues examined, including skeletal muscle (Figure 1A). To determine if FIG4 expression was regulated with maturation of skeletal muscle, we examined protein extracts from skeletal muscle of various postnatal ages (Figure 1B) as well as from differentiating C2C12 myocytes (Figure 1C). We detected no obvious variability in FIG4 levels at different mouse ages or different developmental stages *in vitro*; thus we conclude that FIG4 is a ubiquitously expressed component of skeletal muscle. Of note, we verified that FIG4 expression was absent in *Fig4*-null (that is, *plt*) skeletal muscle as compared to WT littermates (Figure 1D).

### *Fig4*-null myofibers are reduced in size and exhibit ultrastructural abnormalities

We began our analysis by examining both light and electron microscopic features of skeletal muscle from *Fig4*-null mice. We examined muscle at 5 weeks of age, since these mice do not survive beyond 6 weeks of age [32]. We also studied a limited number of animals with expression of a Fig4 cDNA transgene under control of the neuron-specific enolase (NSE) promoter [15]. These animals were examined at 4, 8 and 20 months of age.

**Figure 2:**
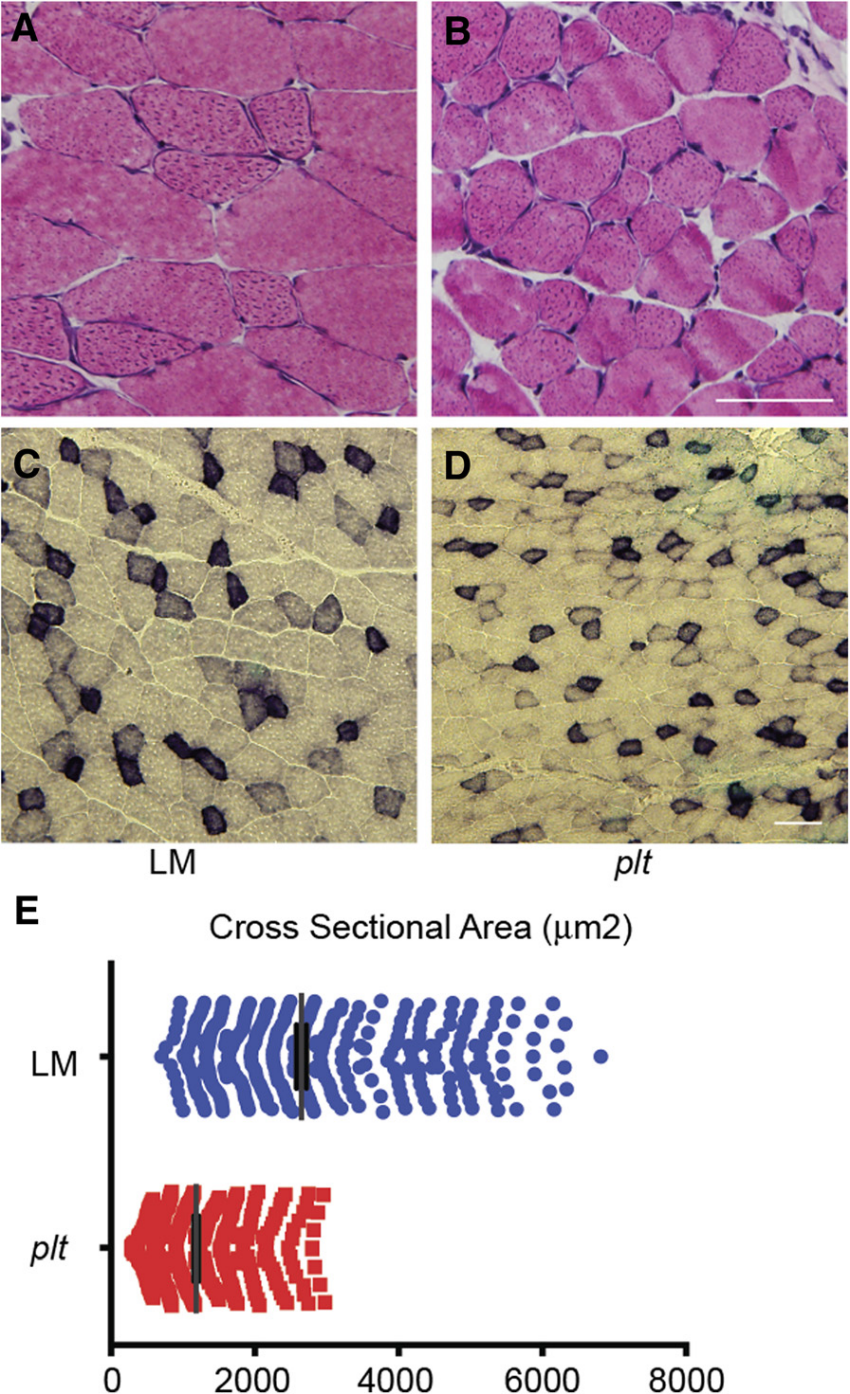
**Histopathological analysis of pale tremor (*plt*) skeletal muscle.** Hematoxylin and eosin staining **(A)** and **(B)** and succinate dehydrogenase staining **(C)** and **(D)** of cryosections from quadriceps of control (wild-type (WT)/littermate (LM)) and *plt* animals. Scale bars = 50 μm. **(E)** Quantitation of myofiber size from quadriceps muscle of 4-week-old *plt* and WT animals. There were significant reductions in myofiber size: 2,649 μm^2^ ±70 for WT vs 1,185 μm^2^ ± 32 for *plt* (*n* = 400 per condition, *P* < 0.0001)**.**

Routine histopathological analysis of quadriceps, gastrocnemius and diaphragm using H&E and SDH stains did not reveal any obvious abnormalities in *plt* skeletal muscle compared to the WT (Figures 2A through 2D). However, the CSA of *plt* myofibers was significantly smaller than that of their WT counterparts. Quantitation of cross-sectional fiber area revealed that *plt* myofibers were 55.3% smaller than WT fibers (*n* = 400 total fibers counted from quadriceps in three mice per condition) (Figure 2E). This reduction in size corresponds with the observation that overall quadriceps weight in *plt* animals was similarly significantly reduced compared to that of age-matched WT littermates (Additional file 1: Figure S1). Of note, overall body weight (which is largely dictated by muscle weight) at 6 weeks of age was also significantly reduced in *plt* mice (Additional file 1: Figure S2).

**Figure 3:**
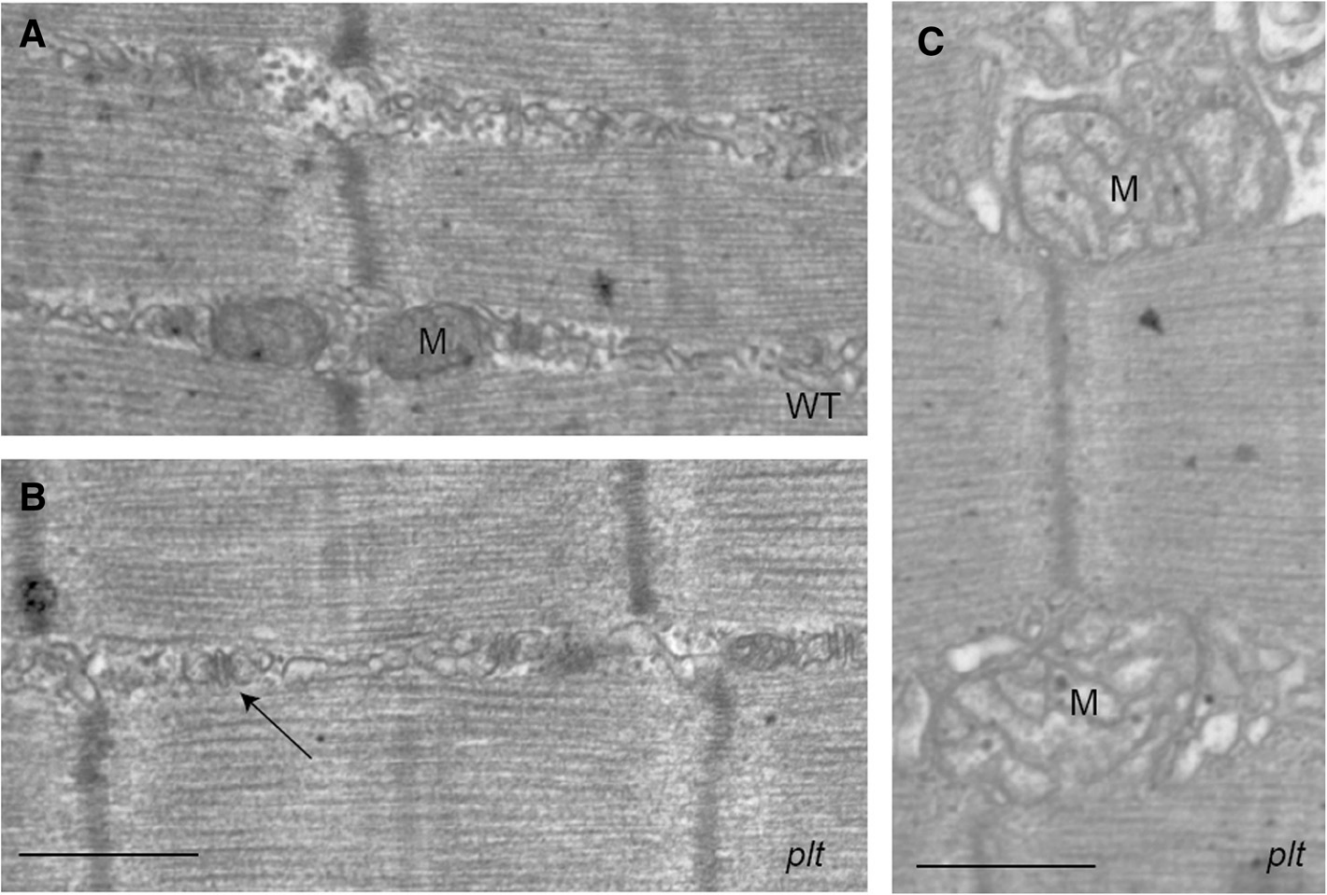
**Ultrastructural analysis of pale tremor (*plt*) skeletal muscle.** Representative photomicrographs from transmission electron microscopic analysis of wild-type/littermate (WT) quadriceps **(A)** and *plt* quadriceps **(B)** and **(C)**. Muscle ultrastructure was generally normal in *plt* muscle **(B)** with evidence of normal triads (arrow). There were infrequently observed areas of abnormalities that particularly included swollen and/or dilated mitochondria (M in **(C)**). Scale bars = 500 nm.

Ultrastructural analyses were performed on quadriceps muscle from age-matched WT and *plt* mice using transmission electron microscopy (*n* = 4 per condition). Overall, muscle ultrastructure in *plt* mice resembled that of controls (Figures 3A and 3B), though swollen and enlarged mitochondria were occasionally observed in muscle from *plt* mice (M in Figure 3C). We did not, however, detect consistent abnormalities in the triad, the location of the EC coupling machinery, in any *plt* mouse examined (arrow in Figure 3B).

### Increased apoptosis in skeletal muscle and primary myocytes from Fig4-deficient mice

**Figure 4:**
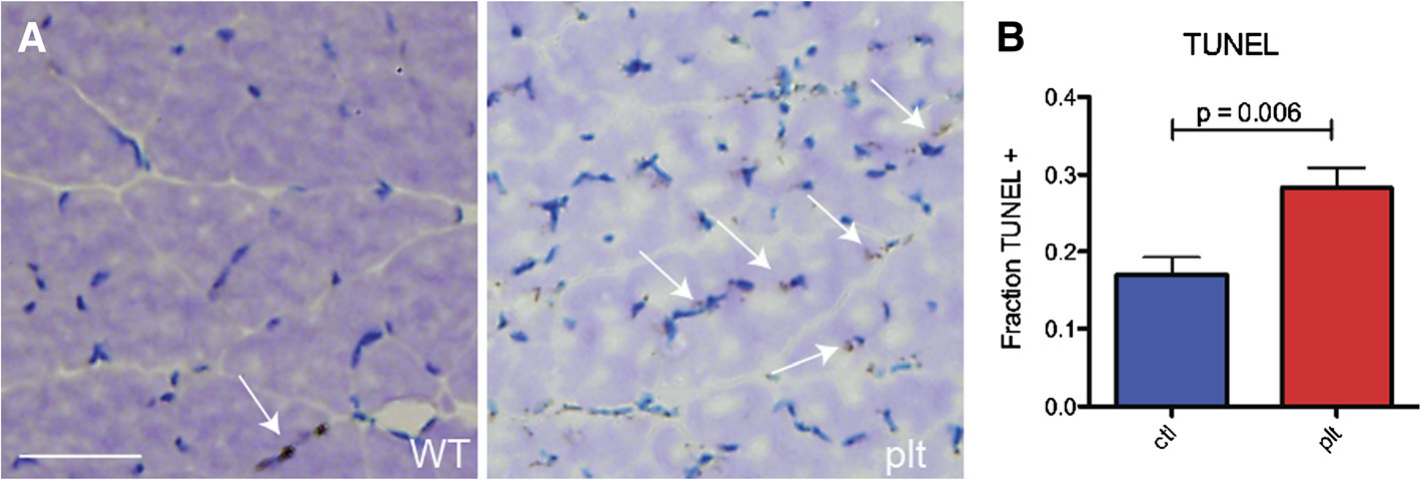
**Increased apoptosis in *plt *skeletal muscle and skeletal myocytes. (A)** Terminal deoxynucleotidyl transferase-mediated dUTP nick end labeling (TUNEL) staining was performed on sections from wild-type (WT) and *plt* skeletal muscle. Arrows point to TUNEL-positive nuclei. There was a qualitative increase in TUNEL-positive fibers in *plt* muscle (experiment repeated with sections from three different mice per condition). **(B)** TUNEL staining was performed on myocytes. There was a significant increase in TUNEL-positive cells in *plt* myocytes vs WT myocytes. Data reflect fraction of 4′,6-diamidino-2-phenylindole-positive nuclei that were also TUNEL-positive. Numbers were 0.28 ± 0.06 for *plt* and 0.17 ± 0.05 for WT (*P* = 0.006). One hundred cells per experiment were counted, and each experiment was repeated five times (once for each culture derived with cells isolated from five different mice per condition).

The combination of reduced muscle fiber size and ultrastructurally abnormal mitochondria led us to examine whether there was increased cell death in *plt* muscle. TUNEL staining of muscle sections revealed few positive cells in the WT tissue but several positive cells in the mutant tissue (Figure 4A). To better evaluate this finding, we studied isolated myocytes from neonatal WT and *plt* mice. As with primary cells from other organ systems of the *plt* mice, cells derived from *plt* muscle demonstrated abundant vacuolization (Additional file 1: Figure S3). We measured cell death in these primary cell cultures using a TUNEL assay. We observed a significant increase in TUNEL-positive cells in *Fig4*-mutant myocytes, consistent with decreased cell survival (Figure 4B). Of note, despite the vacuolization and impaired survival, *Fig4*-null myocytes were able to successfully differentiate into myotubes upon serum withdrawal (data not shown). There was also no change in the ability of *plt* myocytes to proliferate, as determined by bromodeoxyuridine (BrdU) labeling of cells (64% of cells BrdU-positive in control (116 of 182 cells counted) vs 64% of cells positive in *plt* (151 of 235 cells counted).

### FIG4-mutant skeletal muscle has impaired force generation

**Figure 5:**
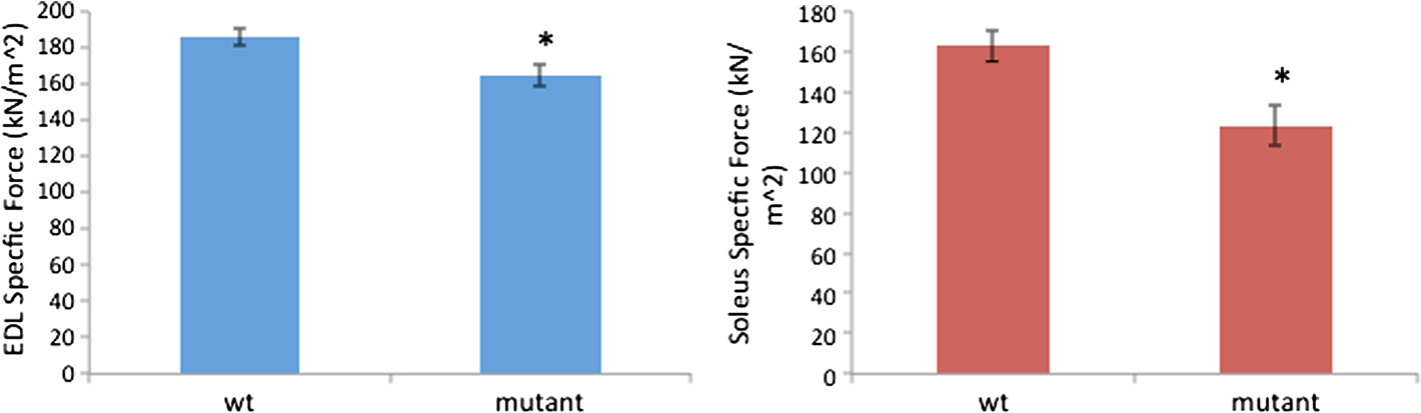
**Diminished force production in pale tremor (*plt*) skeletal muscle.** Specific force (normalized to muscle and body weight) was measured for wild-type/littermate (WT) and *plt* (mutant) skeletal muscle (left panel = extensor digitorum longus, or EDL) (right panel = soleus). Normalized force was significantly reduced in *plt* muscles (*n* = 7 animals tested per condition). *P = 0.043 for EDL and *P* < 0.001 for soleus.

To determine the potential functional effects of *Fig4* deficiency in skeletal muscle, we measured force generation from intact muscle fibers. Testing both EDL and soleus muscles revealed statistically significant reductions in specific force generation (12% to 24% decrease when adjusted for CSA), indicating mild muscle weakness and impaired force generation in the *plt* animals (Figure 5).

### FIG4-mutant myocytes have normal intracellular calcium dynamics

**Figure 6:**
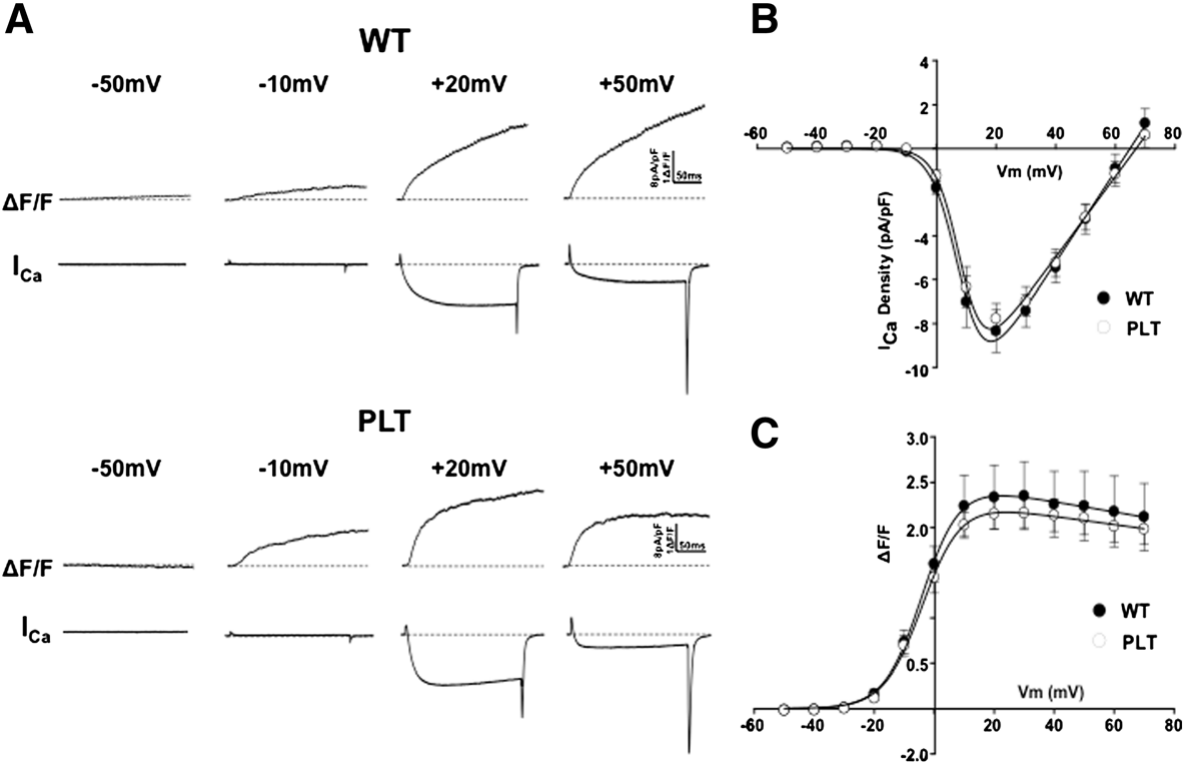
**L-currents and voltage-gated Ca**^**2+ **^**release are unaltered in pale tremor ( *plt *) myotubes. (A)** Representative whole-cell L-type Ca^2+^ currents (*I*_Ca_) and intracellular Ca^2**+**^ transients (ΔF/F) obtained following 200-ms depolarizations to the indicated membrane potentials in myotubes derived from either wild-type (WT) mice (upper panel) or *plt* mice (lower panel). **(B)** and **(C)** Average (±SEM) voltage dependence of peak L-type Ca^2+^ current density **(B)** and intracellular Ca^2+^ transients **(C)** for myotubes derived from either WT mice (filled circles) or *plt* mice (open circles).

Given the previous association between PI(3,5)P_2_ and EC coupling and the fact that FIG4 is a known regulator of PI(3,5)P_2_ levels, we interrogated intracellular calcium dynamics. We used whole-cell voltage patch-clamping of isolated neonatal mouse myocytes to simultaneously measure voltage-gated L-type calcium currents and intracellular calcium transients. We did not detect abnormalities in either orthograde or retrograde coupling or in L-type calcium currents in *plt* myocytes (Figure 6 and Table 1). Taken in conjunction with the normal histological appearance of the triad, these data suggest that loss of *Fig4* is not associated with impaired bidirectional dihydropyridine receptor (DHPR)–RyR1 coupling.

**Table 1:** **Parameters of fitted current-voltage and fluorescence-voltage (∆F/F-V) curves**^**a**^

	***G***_**max **_**(nS/nF)**	***V***_**G1/2 **_**(mV)**	***k***_**G **_**(mV)**	***V***_**REV **_**(mV)**	**(∆F/F)**_**max**_	***V***_**F1/2 **_**(mV)**	***k***_**F**_**(mV)**
WT (n = 11)	219 ± 14	9.8 ± 1.4	5.0 ± 0.5	65.3 ± 3.0	2.3 ± 0.4	−7.8 ± 1.9	4.4 ± 0.2
*plt* (n = 11)	194 ± 15	9.4 ± 1.3	4.0 ± 0.4	66.7 ± 2.7	2.1 ± 0.2	−5.8 ± 1.7	4.6 ± 0.3

^a^*WT* wild type.

### Neuronal expression of *Fig4* in *plt* mice reduces skeletal muscle pathology

**Figure 7:**
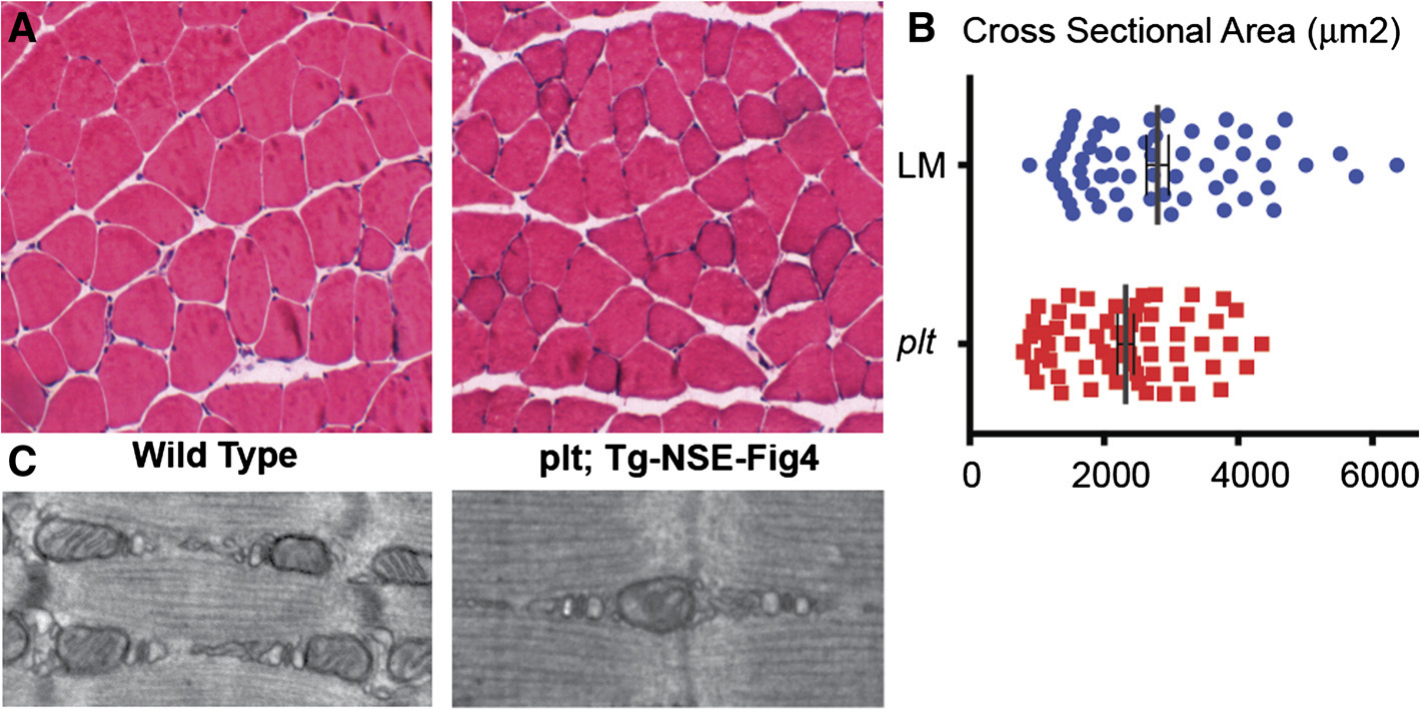
**Transgenic (Tg) neuronal rescue of FIG4 expression largely restores *plt *myofiber size. (A)** Hematoxylin and eosin–stained sections of gastrocnemius muscle from wild-type (WT) and *Fig4*^−/−^/NSE-FIG4 (that is, *plt* with transgenic expression of Fig4 driven by the neuron-specific enolase (NSE) promoter). The *plt* muscle is essentially indistinguishable from WT, with the exception of mild reduction in fiber size. **(B)** Quantification of myofiber size from quadriceps muscles of 8-month-old *Fig4*^−/−^/NSE-FIG4 and WT animals. There was a small but significant reduction in total fiber area in transgenically rescued *plt* muscle: 2,799 μm^2^ ± 165 for WT vs 2,322 μm^2^ ± 121 for *plt* (*n* = 60, *P* = 0.02). **(C)** Transmission electron photomicrographs from tibialis anterior muscle. Normal triads and mitochondria were present in both WT and *plt*/Tg-NSE-Fig4 animals.

We previously reported that expression of FIG4 under the NSE promoter in *plt* animals prevents the development of typical phenotypic abnormalities [15]. In fact, the appearance of the transgenic (Tg) animals is indistinguishable from their WT littermates. We examined skeletal muscle in *Fig4*^−/−^/TgNSE mice at 4, 8 and 20 months of age. There was no obvious difference in histological appearance between WT and *Fig4*^−/−^/TgNSE mice, which is similar to what we observed in the *Fig4*-null mice. Unlike the null mice, however, *Fig4*^−/−^/TgNSE mice exhibited only a very slight alteration in myofiber size (11% smaller than WT; *n* = 5 animals examined per genotype, *P* = 0.02) (Figures 7A and 7B). This reduction was statistically significant and occurred despite the fact that overall weight was the same between WT and *Fig4*^−/−^/TgNSE animals. *Fig4*^−/−^/TgNSE muscle did not display any of the ultrastructural changes seen in the *Fig4*^−/−^ mice (Figure 7C) and did not exhibit evidence of increased apoptosis.

Last, to determine whether muscle function was altered in *Fig4*^−/−^/TgNSE mice, we measured maximum isometric force in soleus and EDL muscles of 18-month-old animals. There was a small (approximately 10%) but statistically insignificant (*P* > 0.1) decline in force generation between *Fig4*^−/−^/TgNSE mice and their WT littermates (Additional file 1: Figure S4). Taking all our data together, we conclude that expression of *Fig4* in neurons is sufficient to correct the development of significant muscle atrophy and programmed cell death in *plt* skeletal muscle, but not to prevent subtle but reproducible changes in myofiber size *in vivo*. Of note, we verified by both RNA (reverse transcriptase polymerase chain reaction) and Western blot analysis that there was no appreciable Fig4 RNA or protein in muscle of *Fig4*^−/−^/TgNSE mice (data not shown and Figure 1D).

### *Fig4* haploinsufficiency does not improve the Mtm1-knockout mouse phenotype

**Figure 8:**
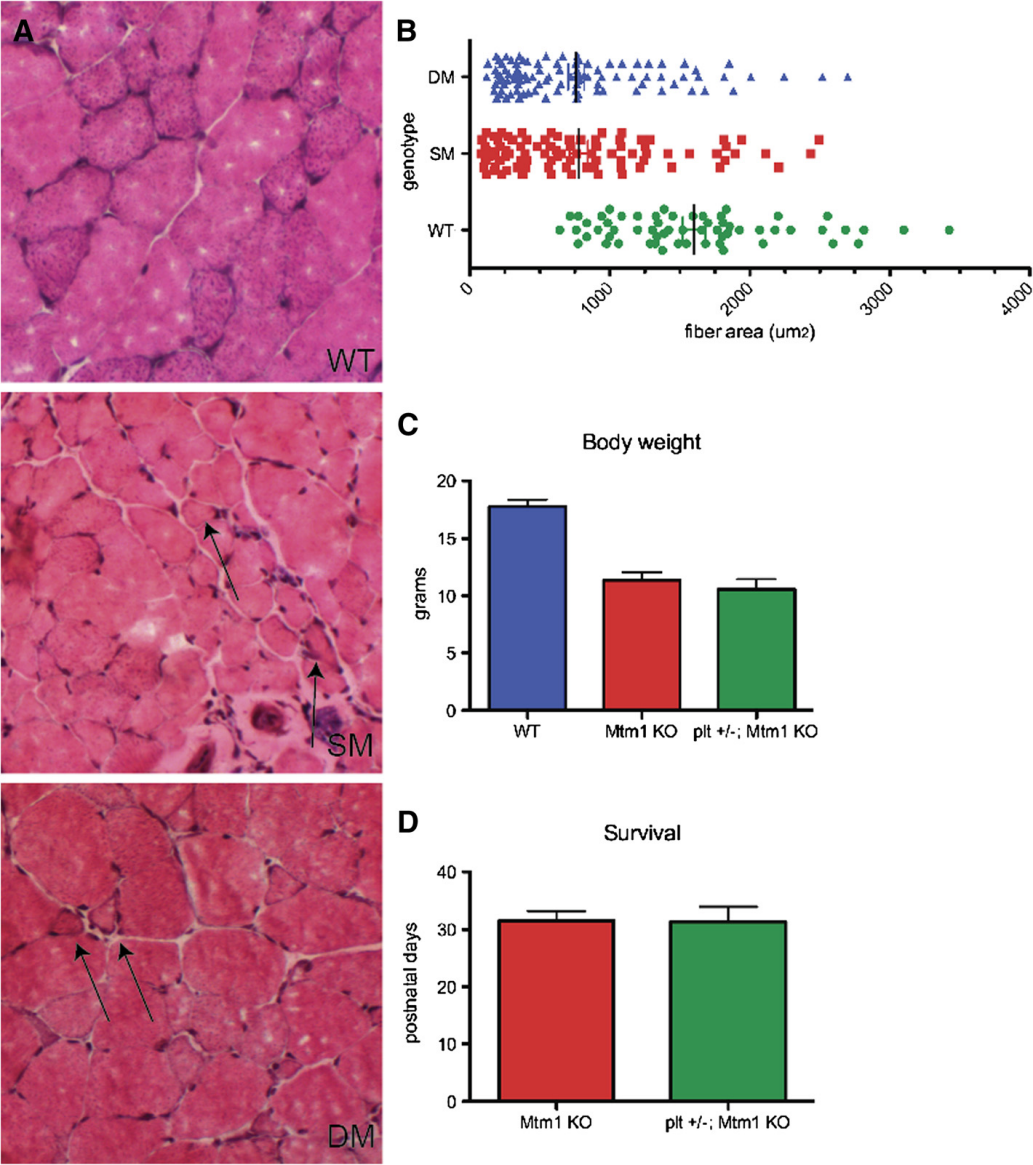
**Fig4 haploinsufficiency does not improve the Mtm1-knockout phenotype. (A)** Hematoxylin and eosin stain of quadriceps muscle from wild-type (WT; *plt*^+/−^) (top), Mtm1-knockout (Mtm1-KO) (SM, middle) and *plt*^+/−^/Mtm1-KO (DM, bottom) animals. There was no obvious difference in the appearance of the muscle or in the number of abnormal fibers (arrows). **(B)** Distribution of fiber area. Fiber area was measured from WT; *plt*^+/−^), single-mutant (SM; Mtm1-KO) and double-mutant (DM; *plt*^+/−^/Mtm1-KO) skeletal muscles. Thirty fibers from each animal were measured (*n* = 4 for SM and DM, *n* =2 for WT). **(C)** Terminal body weight (measured in grams). There was significant reduction in the Mtm1-KO body weight compared to WT. There was no improvement in body weight in *plt*^+/−^/Mtm1-KO animals. Average values were as follows: 17.8 ± 0.6 g for WT (*n* = 8), 11.4 ± 0.7 g for Mtm1-KO (*n* = 9, *P* < 0.0001 compared to WT) and 10.5 ± 0.9 g for *plt*^+/−^/Mtm1-KO (*n* = 5, *P* = 0.48 compared to Mtm1-KO). **(D)** Haploinsufficiency of Fig4 did not provide any survival benefit. Average survival was 32 ± 2 days for Mtm1-KO (*n* = 9) and 31 ± 3 days for *plt*^+/−^/Mtm1-KO (*n* = 5).

There is a complex interplay between the enzymes that regulate phosphoinositides. One potential avenue for treatment of diseases related to PIP dysregulation is manipulation of other enzyme levels. This point has been demonstrated for the neurological abnormalities associated with *Mtmr2* mutation [30]. Mice with a recessive mutation in *Mtmr2* exhibit peripheral neuropathy, and aspects of this neuropathy are reversed in the setting of *Fig4* haploinsufficiency. Since MTM1 and MTMR2 are highly homologous, we evaluated the effect of haploinsufficiency of *Fig4* (in *plt*^+/−^ mice) on the severe muscle phenotype in knockout mice lacking expression of *Mtm1*. Generation of *Fig4*^+/−^/*Mtm1*^*–/Y*^ male mice revealed no difference from *Mtm1*^*–/Y*^ mice, with comparable impairments of weight gain, motor function and survival and no statistically significant difference in weight or survival (Figures 8A and 8B).

By histopathological analysis, we demonstrated similar alterations between *Mtm1*^–/*Y*^ mice and *Mtm1*^–/*Y*^/*Fig4*^*+/−*^ mice. We quantitated myofiber size because size is correlated with disease severity in patients with MTM [36]. There was no clear difference in this parameter (Figure 8B, consistent with the observed lack of functional improvement in the *Fig4*^+/–^/*Mtm1*^*–/Y*^ animals. In all, haploinsufficiency of *Fig4* did not appear to alter the *Mtm1* phenotype in any meaningful way.

## Discussion

FIG4 exhibits an important role in central and peripheral nervous system development and homeostasis. The consequences of FIG4 deficiency on other organ systems are less well delineated. In this study, we defined the consequences of *Fig4*-null mutation on skeletal muscle. We found that (1) global inactivation of *Fig4* in the *plt* mouse is associated with myofiber atrophy and/or hypotrophy, increased apoptosis and diminished specific force production; (2) loss of *Fig4* does not alter the structure, EC coupling apparatus or bidirectional DHPR–RyR1 coupling; and (3) reexpression of *Fig4* in neurons significantly reduces all observed muscle abnormalities. The implications of these findings are discussed below.

Perhaps the most striking aspect of this study is the fact that *Fig4* mutation did not result in a more deleterious direct effect on muscle development and function. In neurons and glia, loss of FIG4 results in severe structural and functional consequences. Given the previously recognized importance of phosphoinositide regulation in skeletal muscle [26,27], we predicted that *plt* mice would exhibit pronounced changes in muscle structure and function. However, effects of *Fig4* ablation on muscle were minimal and largely rescued following neuron-specific restoration of FIG4 expression (*Fig4*^*−/−*^/TgNSE mice). Muscle alterations found to persist in *Fig4*^*−/−*^/TgNSE mice (for example, 11% reduction in fiber size) could be explained by either a minor role for FIG4 expression in muscle fiber size determination or a small amount of residual neurogenic atrophy. It is of interest to note the dichotomy between the relatively normal appearance of adult *plt* muscle *in situ* and the extensive vacuolarization of *plt* myocytes *in vitro*. Similar effects of culturing have been observed in other cell types, including fibroblasts [12] and osteoblasts (unpublished manuscript, Lenk GM and Meisler MH). The explanation for this difference is unclear and requires further experimentation.

There are several potential explanations for the lack of a more severe phenotype in skeletal muscle of *Fig4*-null mice. First of all, the partial reduction of PI(3,5)P_2_ likely caused by loss of *Fig4* may not be enough to result in significant consequences for muscle development and function. Alternatively, a different PIKfyve protein complex may compensate for the loss of FIG4 to provide sufficient levels of PI(3,5)P_2_ to maintain myofiber homeostasis. A third possibility is that PI(3,5)P_2_ may be generated in muscle by a different or complementary pathway. For example, a three-position kinase may generate PI(3,5)P_2_ from PI(5)P. One current barrier to attempting to distinguish these possibilities is the lack of suitable approaches to measure PI(3,5)P_2_ in whole tissues such as skeletal muscle. Development of techniques to aid in measuring PI(3,5)P_2_*in situ* are required to answer these questions more definitively.

Regardless of the explanation, the fact that there is little, if any, muscle-cell autonomous phenotype in *Fig4*-null animals indicates that *FIG4* mutations are unlikely to result in primary muscle disease. However, significant secondary neurogenically mediated myopathic features, such as those observed in *plt* mice, including reduced muscle fiber CSA and specific force generation, suggest that skeletal muscle changes may contribute to disease pathogenesis. In other words, the myopathic changes described herein may influence disease severity in patients with Charcot-Marie-Tooth disease type 4J and other disorders caused by *FIG4* gene mutation.

The lack of impairment in the structure of the EC coupling apparatus or of bidirectional triad coupling in *Fig4*-null mice was unexpected. Data from the MTMR14-knockout mice support the hypothesis that increased levels of PI(3,5)P_2_ impair calcium release from the ryanodine receptor (the core component of the EC apparatus), though the underlying mechanism is not clear. Our data imply that reduced levels of PI(3,5)P_2_ do not acutely impair voltage-gated triad calcium release. Furthermore, the chronic loss of FIG4 from muscle (with the potential implication of chronically reduced PI(3,5)P_2_ levels) does not alter the ultrastructural appearance of the triad (that is, the location of the EC coupling machinery). Thus, a requirement for normal levels of PI(3,5)P_2_ for EC coupling seems unlikely, though the present data do not completely exclude this possibility. In addition to more direct interrogation of EC coupling in FIG4-deficient mice, another potential future direction to address this issue would be to assess the impact on EC coupling of muscle-specific knockout of PIKfyve, the kinase required for PI(3,5)P_2_ generation. Again, however, this would necessitate confirmation of a specific reduction in PI(3,5)P_2_ levels in skeletal muscle.

The final significant observation in this study is that reduced *Fig4* expression via *plt* haploinsufficiency does not significantly alter the phenotype of *Mtm1*-knockout mice. Of note, the *MTMR2* gene encodes a protein that is highly homologous to *MTM1*[8]. In addition, we previously demonstrated that zebrafish *mtmr2* functionally compensates (at least in part) for loss of *mtm1*, suggesting that MTMR2 and MTM1 are functionally quite similar [37]. However, in contrast to our findings in *Mtm1-*null mice, Bolino and colleagues found that *plt* haploinsufficiency rescued neuropathy in *Mtmr2*-knockout animals [30]. The reasons why reduction of FIG4 levels improved the MTMR2-related neuropathology, but not the muscle pathology, seen in *Mtm1-*null mice are not clear. This distinction may provide another indication of the nonessential role of FIG4 in skeletal muscle or may reflect different quantitative requirements for PI(3,5)P_2_ in neurons and muscle or specific differences between mammalian MTMR2 and MTM1.

## Conclusions

We present data demonstrating that FIG4 is required for muscle function but is dispensable for muscle development. In addition, most abnormalities associated with Fig4 mutation appears to be secondary to the severe neuropathy documented in *plt* mice. Our results do not support a role for FIG4 in EC coupling. Future experiments are needed to more firmly establish the relationship between PI(3,5)P_2_ and EC coupling.

## Abbreviations

DHPR: Dihydropyridine receptor; EC coupling: Excitation–contraction coupling; NSE: Neuron-specific enolase; PI(3)P: Phosphatidylinositol 3-phosphate; PI(3,5)P2: Phosphatidylinositol 3,5-bisphosphate; PIP: Phosphatidylinositol phosphate; plt: Pale tremor mouse; RyR1: Skeletal muscle ryanodine receptor 1; TUNEL: Terminal deoxynucleotidyl transferase-mediated dUTP nick end labeling.

## Competing interests

The authors declare that they have no competing interests.

## Authors’ contributions

AR performed the majority of experiments, aided with data interpretation and helped generate the manuscript. XL, DW and MB helped perform the experiments. GL helped with mouse husbandry and manuscript generation. SVB performed and interpreted muscle force experimentation. LG and RTD designed, performed, analyzed and interpreted the myotube voltage-clamp experiments. MM aided with experimental design, data interpretation and manuscript generation. JJD conceived the project, helped with all data interpretation and generated the manuscript. All authors read and approved the final manuscript.

## Supplementary Material

Additional file 1: Figure S1 Reduced muscle mass in *plt* animals. **Figure S2.** Reduced body mass in *plt* animals. **Figure S3.** Vacuoles are present in *plt* myocytes. **Figure S4.** Muscle force is restored to normal in *Fig4 -/-;* Tg;NSE mice. Click here for file

## References

[B1] Di Paolo G De Camilli P Phosphoinositides in cell regulation and membrane dynamics Nature 2006 443 651 657 10.1038/nature05185 17035995

[B2] De Camilli P Emr SD McPherson PS Novick P Phosphoinositides as regulators in membrane traffic Science 1996 271 1533 1539 10.1126/science.271.5255.1533 8599109

[B3] Ho CY Alghamdi TA Botelho RJ Phosphatidylinositol-3,5-*bis*phosphate: no longer the poor PIP_2_ Traffic 2012 13 1 8 10.1111/j.1600-0854.2011.01246.x 21736686

[B4] Zolov SN Bridges D Zhang Y Lee WW Riehle E Verma R Lenk GM Converso-Baran K Weide T Albin RL Saltiel AR Meisler MH Russell MW Weisman LS In vivo, Pikfyve generates PI(3,5)P_2_, which serves as both a signaling lipid and the major precursor for PI5P Proc Natl Acad Sci USA 2012 109 17472 17477 10.1073/pnas.1203106109 23047693 PMC3491506

[B5] Tosch V Rohde HM Tronchère H Zanoteli E Monroy N Kretz C Dondaine N Payrastre B Mandel JL Laporte J A novel PtdIns3*P* and PtdIns(3,5)*P*_2_ phosphatase with an inactivating variant in centronuclear myopathy Hum Mol Genet 2006 15 3098 3106 10.1093/hmg/ddl250 17008356

[B6] Hakim S Bertucci MC Conduit SE Vuong DL Mitchell CA Inositol polyphosphate phosphatases in human disease Curr Top Microbiol Immunol 2012 362 247 314 10.1007/978-94-007-5025-8_12 23086422

[B7] Dyson JM Fedele CG Davies EM Becanovic J Mitchell CA Phosphoinositide phosphatases: just as important as the kinases Subcell Biochem 2012 58 215 279 10.1007/978-94-007-3012-0_7 22403078

[B8] Robinson FL Dixon JE Myotubularin phosphatases: policing 3-phosphoinositides Trends Cell Biol 2006 16 403 412 10.1016/j.tcb.2006.06.001 16828287

[B9] Jin N Chow CY Liu L Zolov SN Bronson R Davisson M Petersen JL Zhang Y Park S Duex JE Goldowitz D Meisler MH Weisman LS VAC14 nucleates a protein complex essential for the acute interconversion of PI3P and PI(3,5)P_2_ in yeast and mouse EMBO J 2008 27 3221 3234 10.1038/emboj.2008.248 19037259 PMC2600653

[B10] Duex JE Tang F Weisman LS The Vac14p–Fig4p complex acts independently of Vac7p and couples PI3,5P_2_ synthesis and turnover J Cell Biol 2006 172 693 704 10.1083/jcb.200512105 16492811 PMC2063702

[B11] Gary JD Sato TK Stefan CJ Bonangelino CJ Weisman LS Emr SD Regulation of Fab1 phosphatidylinositol 3-phosphate 5-kinase pathway by Vac7 protein and Fig4, a polyphosphoinositide phosphatase family member Mol Biol Cell 2002 13 1238 1251 10.1091/mbc.01-10-0498 11950935 PMC102265

[B12] Chow CY Zhang Y Dowling JJ Jin N Adamska M Shiga K Szigeti K Shy ME Li J Zhang X Lupski JR Weisman LS Meisler MH Mutation of *FIG4* causes neurodegeneration in the pale tremor mouse and patients with CMT4J Nature 2007 448 68 72 10.1038/nature05876 17572665 PMC2271033

[B13] Winters JJ Ferguson CJ Lenk GM Giger-Mateeva VI Shrager P Meisler MH Giger RJ Congenital CNS hypomyelination in the *Fig4* null mouse is rescued by neuronal expression of the PI(3,5)P_2_ phosphatase *Fig4* J Neurosci 2011 31 17736 17751 10.1523/JNEUROSCI.1482-11.2011 22131434 PMC3711465

[B14] Ferguson CJ Lenk GM Meisler MH Defective autophagy in neurons and astrocytes from mice deficient in PI(3,5)P_2_ Hum Mol Genet 2009 18 4868 4878 10.1093/hmg/ddp460 19793721 PMC2778378

[B15] Ferguson CJ Lenk GM Jones JM Grant AE Winters JJ Dowling JJ Giger RJ Meisler MH Neuronal expression of *Fig4* is both necessary and sufficient to prevent spongiform neurodegeneration Hum Mol Genet 2012 21 3525 3534 10.1093/hmg/dds179 22581779 PMC3406753

[B16] Ikonomov OC Sbrissa D Fligger J Delvecchio K Shisheva A ArPIKfyve regulates Sac3 protein abundance and turnover: disruption of the mechanism by Sac3^I41T^ mutation causing Charcot-Marie-Tooth 4J disorder J Biol Chem 2010 285 26760 26764 10.1074/jbc.C110.154658 20630877 PMC2930674

[B17] Lenk GM Ferguson CJ Chow CY Jin N Jones JM Grant AE Zolov SN Winters JJ Giger RJ Dowling JJ Weisman LS Meisler MH Pathogenic mechanism of the FIG4 mutation responsible for Charcot-Marie-Tooth disease CMT4J PLoS Genet 2011 7 e1002104 10.1371/journal.pgen.1002104 21655088 PMC3107197

[B18] Nicholson G Lenk GM Reddel SW Grant AE Towne CF Ferguson CJ Simpson E Scheuerle A Yasick M Hoffman S Blouin R Brandt C Coppola G Biesecker LG Batish SD Meisler MH Distinctive genetic and clinical features of CMT4J: a severe neuropathy caused by mutations in the PI(3,5)P_2_ phosphatase *FIG4* Brain 2011 134 1959 1971 10.1093/brain/awr148 21705420 PMC3122378

[B19] Chow CY Landers JE Bergren SK Sapp PC Grant AE Jones JM Everett L Lenk GM McKenna-Yasek DM Weisman LS Figlewicz D Brown RH Meisler MH Deleterious variants of *FIG4*, a phosphoinositide phosphatase, in patients with ALS Am J Hum Genet 2009 84 85 88 10.1016/j.ajhg.2008.12.010 19118816 PMC2668033

[B20] Romero-Suarez S Shen J Brotto L Hall T Mo C Valdivia HH Andresen J Wacker M Nosek TM Qu CK Brotto M Muscle-specific inositide phosphatase (MIP/MTMR14) is reduced with age and its loss accelerates skeletal muscle aging process by altering calcium homeostasis Aging (Albany NY) 2010 2 504 513 20817957 10.18632/aging.100190 PMC2954041

[B21] Laporte J Bedez F Bolino A Mandel JL Myotubularins, a large disease-associated family of cooperating catalytically active and inactive phosphoinositides phosphatases Hum Mol Genet 2003 12 Spec No 2 R285 R292 12925573 10.1093/hmg/ddg273

[B22] Gibbs EM Feldman EL Dowling JJ The role of MTMR14 in autophagy and in muscle disease Autophagy 2010 6 819 820 10.4161/auto.6.6.12624 20595810

[B23] Hnia K Vaccari I Bolino A Laporte J Myotubularin phosphoinositide phosphatases: cellular functions and disease pathophysiology Trends Mol Med 2012 18 317 327 10.1016/j.molmed.2012.04.004 22578719

[B24] Pierson CR Dulin-Smith AN Durban AN Marshall ML Marshall JT Snyder AD Naiyer N Gladman JT Chandler DS Lawlor MW Buj-Bello A Dowling JJ Beggs AH Modeling the human *MTM1* p.R69C mutation in murine *Mtm1* results in exon 4 skipping and a less severe myotubular myopathy phenotype Hum Mol Genet 2012 21 811 825 10.1093/hmg/ddr512 22068590 PMC3263994

[B25] Amoasii L Hnia K Laporte J Myotubularin phosphoinositide phosphatases in human diseases Curr Top Microbiol Immunol 2012 362 209 233 10.1007/978-94-007-5025-8_10 23086420

[B26] Dowling JJ Low SE Busta AS Feldman EL Zebrafish MTMR14 is required for excitation–contraction coupling, developmental motor function and the regulation of autophagy Hum Mol Genet 2010 19 2668 2681 10.1093/hmg/ddq153 20400459 PMC2883342

[B27] Al-Qusairi L Weiss N Toussaint A Berbey C Messaddeq N Kretz C Sanoudou D Beggs AH Allard B Mandel JL Laporte J Jacquemond V Buj-Bello A T-tubule disorganization and defective excitation–contraction coupling in muscle fibers lacking myotubularin lipid phosphatase Proc Natl Acad Sci USA 2009 106 18763 18768 10.1073/pnas.0900705106 19846786 PMC2773964

[B28] Shen J Yu WM Brotto M Scherman JA Guo C Stoddard C Nosek TM Valdivia HH Qu CK Deficiency of MIP/MTMR14 phosphatase induces a muscle disorder by disrupting Ca^2+^ homeostasis Nat Cell Biol 2009 11 769 776 10.1038/ncb1884 19465920 PMC2693472

[B29] Touchberry CD Bales IK Stone JK Rohrberg TJ Parelkar NK Nguyen T Fuentes O Liu X Qu CK Andresen JJ Valdivia HH Brotto M Wacker MJ Phosphatidylinositol 3,5-bisphosphate (PI(3,5)P_2_) potentiates cardiac contractility via activation of the ryanodine receptor J Biol Chem 2010 285 40312 40321 10.1074/jbc.M110.179689 20947503 PMC3001011

[B30] Vaccari I Dina G Tronchère H Kaufman E Chicanne G Cerri F Wrabetz L Payrastre B Quattrini A Weisman LS Meisler MH Bolino A Genetic interaction between MTMR2 and FIG4 phospholipid phosphatases involved in Charcot-Marie-Tooth neuropathies PLoS Genet 2011 7 e1002319 10.1371/journal.pgen.1002319 22028665 PMC3197679

[B31] Bolino A Bolis A Previtali SC Dina G Bussini S Dati G Amadio S Del Carro U Mruk DD Feltri ML Cheng CY Quattrini A Wrabetz L Disruption of *Mtmr2* produces CMT4B1-like neuropathy with myelin outfolding and impaired spermatogenesis J Cell Biol 2004 167 711 721 10.1083/jcb.200407010 15557122 PMC2172586

[B32] Lenk GM Meisler MH Mouse models with defects in PI(3,5)P_2_ and impaired endolysosome function Methods Enzymolin press 10.1016/B978-0-12-397926-1.00014-7 PMC4059992 24359958

[B33] Brooks SV Faulkner JA Contractile properties of skeletal muscles from young, adult and aged mice J Physiol 1988 404 71 82 3253447 10.1113/jphysiol.1988.sp017279 PMC1190815

[B34] Avila G O’Brien JJ Dirksen RT Excitation–contraction uncoupling by a human central core disease mutation in the ryanodine receptor Proc Natl Acad Sci USA 2001 98 4215 4220 10.1073/pnas.071048198 11274444 PMC31205

[B35] Avila G Dirksen RT Functional effects of central core disease mutations in the cytoplasmic region of the skeletal muscle ryanodine receptor J Gen Physiol 2001 118 277 290 10.1085/jgp.118.3.277 11524458 PMC2229502

[B36] Pierson CR Agrawal PB Blasko J Beggs AH Myofiber size correlates with *MTM1* mutation type and outcome in X-linked myotubular myopathy Neuromuscul Disord 2007 17 562 568 10.1016/j.nmd.2007.03.010 17537630 PMC2043149

[B37] Dowling JJ Vreede AP Low SE Gibbs EM Kuwada JY Bonnemann CG Feldman EL Loss of myotubularin function results in T-tubule disorganization in zebrafish and human myotubular myopathy PLoS Genet 2009 5 e1000372 10.1371/journal.pgen.1000372 19197364 PMC2631153

