# Supplementary material for: Murine Fig4 is dispensable for muscle development but required for muscle function
Source: Skelet Muscle. 2013 Sep 1;3:21. doi: 10.1186/2044-5040-3-21 (PMC3844516; doi:10.1186/2044-5040-3-21)
Supplement: Additional file 1: Figure S1 — Reduced muscle mass in plt animals. Figure S2. Reduced body mass in plt animals. Figure S3. Vacuoles are present in plt myocytes. Figure S4. Muscle force is restored to normal in Fig4 -/-; Tg;NSE mice. [file 2044-5040-3-21-S1.docx]

SUPPLEMENTAL FIGURES


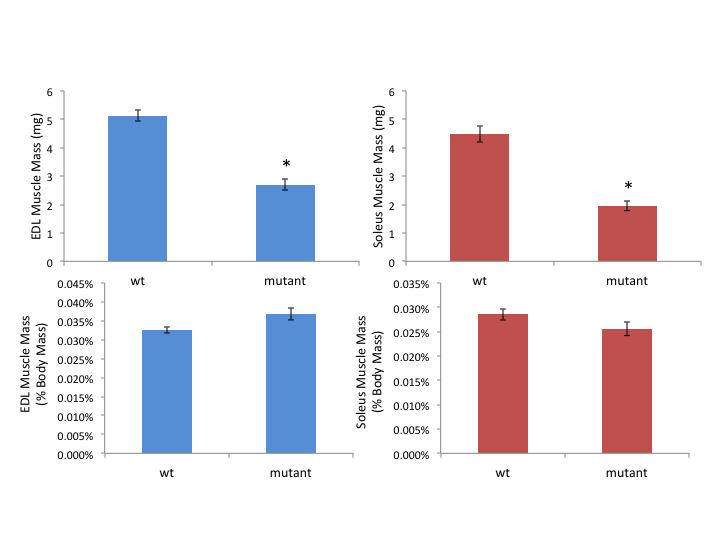


**Supplemental Figure 1: Reduced muscle mass in *plt* animals**

Muscle from wild type (WT) and *plt* (mutant) animals was weighed just prior to muscle force measurements. There was a significant reduction in average muscle mass in *plt* animals in both the extensor digitorum longus (EDL, left) and the soleus (right).


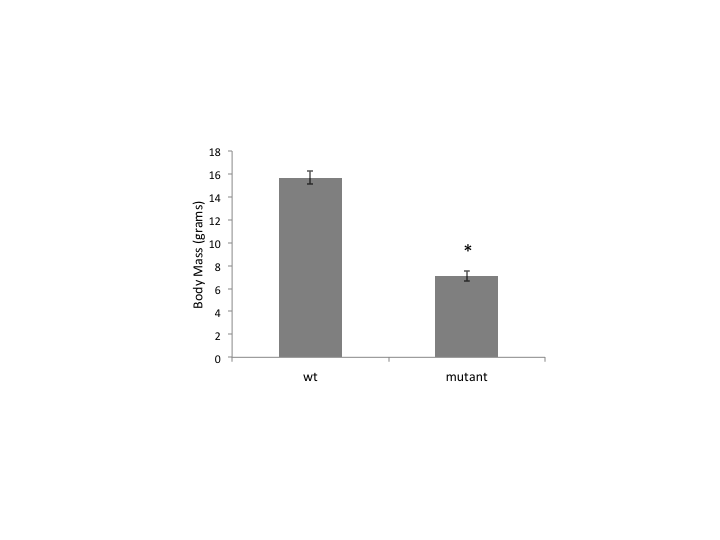
**Supplemental Figure 2: Reduced body mass in *plt* animals**

Wild type (WT) and plt (mutant) animals were weighed just prior to muscle force measurements. There was a significant reduction in average body mass in *plt* animals.


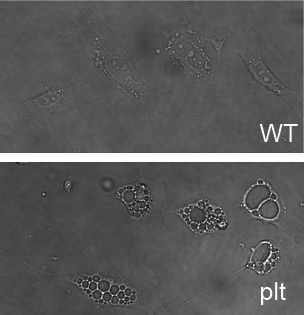


**Supplemental Figure 3: Vacuoles are present in *plt* myocytes**

Phase contrast representative photomicrograph of WT and *plt* skeletal myoblasts. Note the extensive vacuolization in the *plt* myoblasts.


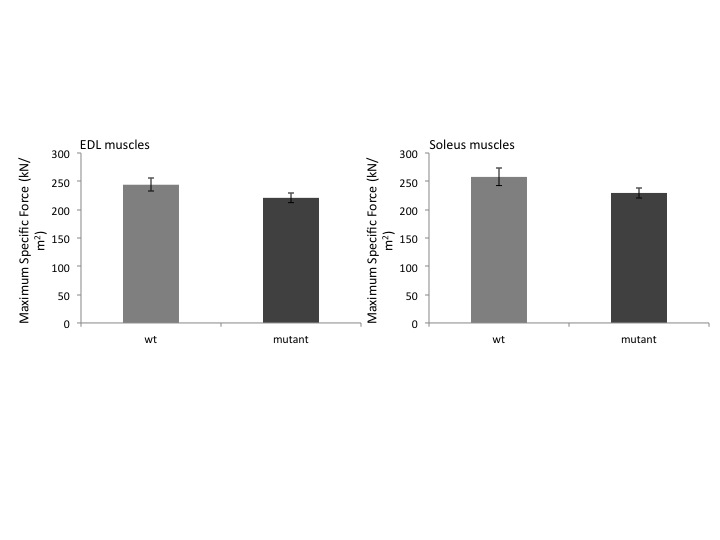


**Supplemental Figure 4: Muscle force is restored to normal in *Fig4 -/-;* Tg;NSE mice**

Maximum isometric specific force (normalized to total muscle fiber cross-sectional area) was measured in wild type (wt) and Fig4 -/-; Tg;NSE-Fig4 (mutant) animals (n = 3 animals per condition). There was no difference in force generation observed between the two conditions. Specific results are as follows: EDL muscles (p = 0.13) wt = 244 ± 11 kN/m^2^; mutant = 221 ± 8 kN/m^2^; soleus muscles (p = 0.17): wt = 258 ± 15 kN/m^2^; mutant = 230 ± 9 kN/m^2^.
